# Clinical and Paraclinical Considerations Regarding ki67’s Role in the Management of Differentiated Thyroid Carcinoma—A Literature Review

**DOI:** 10.3390/medicina60050769

**Published:** 2024-05-07

**Authors:** Claudiu Peștean, Alexandru Pavel, Doina Piciu

**Affiliations:** 1 Faculty of Medicine, Iuliu Hațieganu University of Medicine and Pharmacy, 400012 Cluj-Napoca, Romania; 2 Prof. Dr. Ion Chiricuță Institute of Oncology, Department of Nuclear Medicine, 400015 Cluj-Napoca, Romania; 3 Affidea CT Clinic, 400015 Cluj-Napoca, Romania; 4 Emergency Clinical County Hospital, 400347 Cluj-Napoca, Romania

**Keywords:** ki67, proliferation index, differentiated thyroid carcinoma, prognosis

## Abstract

*Background and Objectives*: The ki67 nuclear protein is a tool for diagnosis and prognosis in oncology that is used to evaluate cell proliferation. Differentiated thyroid carcinoma is usually a slow-growing neoplasm, the most common type being the papillary form. Some clinical and pathological aspects may predict aggressive behaviour. There are reported cases of recurrence without clinico-pathological findings of aggressiveness. To obtain better predictions of the disease outcome in thyroid carcinoma, many immunohistochemical markers have been studied. The aim of this narrative literature review is to identify the benefits that ki67 may add to the management of patients with differentiated thyroid carcinoma, according to the latest evidence. *Materials and Methods:* We performed a search on the PubMed and Google Scholar databases using controlled vocabulary and keywords to find the most suitable published articles. A total number of sixty-eight items were identified, and five other articles were selected from other sources. After refining the selection, the inclusion criteria and exclusion criteria were applied, and a total number of twenty-nine articles were included in this literature review. *Results and Discussion:* The studies consist of retrospective studies (89.66%), case reports (6.9%) and literature reviews (3.45%), evaluating the role, implications and other parameters of ki67 as a diagnostic and/or prognostic tool. The statistical correlations between ki67 and other features were systematized as qualitative results of this review in order to improve the treatment strategies presented in the included articles. *Conclusions:* The included studies present converging data regarding most of the aspects concerning ki67. The ki67 proliferation index is a diagnostic/prognostic tool of interest in differentiated thyroid carcinoma and a good predictor of disease-free survival, disease recurrence and metastatic development. Prospective studies on large cohorts may add value for ki67 as a specific tool in the management strategy of differentiated thyroid carcinoma.

## 1. Introduction

When significant genetic and epigenetic alterations occur in a single cell of the human body, it may start to divide and expand uncontrollably. This process could lead to neoplastic transformation characterised by an aberrant cell proliferation process affecting the body systems and organs at a physiological and morphological level. Even though cancer is not entirely defined by aberrant cell proliferation, this aspect is one of the main targets aimed at by diagnostic, prognostic and treatment strategies. Among the principles of cancer treatment strategies, an inverse relationship is demonstrated between tumour size and therapeutic success. There are clinical situations described in the literature where tiny tumours grow faster than the same type of cancer with a larger mass and the same cell type [1]. The type of therapy is essential in cancer treatment but not sufficient. The understanding of cancer biology and cancer genetics to achieve the best results is essential.

Among all the therapeutic aspects approached by the scientists and clinicians fighting against cancer, the aspects concerning tumour cell proliferation, tumour growth, the immuno-histological particularities related to ki67 (originally marker of proliferation Kiel 67, sometimes named MKI67) expression and the malignant cellular mitotic rate play an important role in the aggressiveness assessment and aggressiveness prognosis. These features are widely used as proliferation indicators [2,3].

The ki67 nuclear protein (pki67) is a tool successfully used for the diagnosis and prognosis in oncology, proving to be beneficial in cell proliferation evaluation. Also, ki67 is a therapeutic target in some pathologies like cervical cancer and breast, lung, bladder, urothelial, upper urinary tract carcinomas and lymphomas [3,4].

Differentiated thyroid carcinoma (DTC) is a slow-growing neoplasm. The most common type of DTC is the papillary form, corresponding to more than 80% of cases [5]. It can metastasize to cervical lymph nodes without affecting the survival rate, which can be up to 98% at 5 years and 93% at 10 years, with a recurrence rate of about 28% [5]. Some clinical and pathological aspects may predict the aggressive tumour behaviour of the differentiated thyroid cancers. These features are the patient’s age, tumour size and presence of tumour invasion, distant metastases and dedifferentiation of the tumour [6]. Some cases of recurrence have been reported without having these aggressiveness clinico-pathological findings [5]. It is obvious that any valuable predictive factor aside from the clinico-pathological features already described would be useful to more accurately anticipate the patient outcome and, therefore, to establish the most pertinent therapeutic strategy for the patient. The immunohistochemical methods have been used in differentiated thyroid carcinoma cases as a new tool to improve the ability to predict the recurrence risk based on the tumour aggressiveness estimation. Ki67 seems to be one of the most promising immunohistochemical markers assessing cell proliferation and, thus, tumour aggressiveness [5].

The thyroid cancer (TC) incidence has increased consistently in recent decades, being the most common endocrine malignancy, occupying fifth place in terms of the statistics covering the diagnosed cancers among women, accounting for approximately 2.1% of all the cancer diagnoses in the world, with 77% attributed to female patients [7,8,9,10].

In light of the 2022 WHO (World Health Organization) histological classification regarding thyroid neoplasms, the differentiated types of TC comprise low-risk neoplasms and malignant neoplasms.

The low-risk neoplasm category includes the following:

- non-invasive follicular thyroid neoplasms with papillary-like nuclear features;
- thyroid tumours of uncertain malignant potential;
- follicular tumours of uncertain malignant potential;
- well-differentiated tumours of uncertain malignant potential.

The malignant neoplasms included as differentiated are the following:

- follicular thyroid carcinoma (FTC);
- invasive encapsulated follicular variant papillary thyroid carcinoma (PTC);
- papillary thyroid carcinoma (classic subtype, encapsulated classic subtype, infiltrative follicular subtype, diffuse sclerosing subtype, solid/trabecular subtype, Warthin-like subtype, oncocytic subtype, clear cell subtype, spindle cell subtype, papillary thyroid carcinoma with fibromatosis/fasciitis-like/desmoid-type stroma, tall cell subtype, hobnail subtype and columnar cell subtype);
- oncocytic carcinoma (OC) of the thyroid;
- differentiated high-grade thyroid carcinomas (as a subtype of differentiated high-grade follicular-derived carcinoma) [11].

### The Role of ki67 in the Tumour Proliferation Assessment

Moreover, ki67 is a non-histone protein, a nuclear antigen found only in the dividing cells in the G1, S, G2 and M-cell phases. The ki67 expression peaks in the early phases of mitosis, but the levels decrease dramatically during the later phases (anaphase and telophase), and ki67 is absent in the G0 phase of resting cells. Its expression is significantly higher in neoplasms than in normal cells, the ki67 levels tending to increase with the decrease in cell differentiation [2,12]. The half-life of ki67 is about 1.5 h, and the precise level during the cell phase is maintained by an equilibrium between synthesis and degradation [2,13]. As a diagnostic tool used as a proliferation indicator, the ki67 index has good predictive value since it estimates tumour aggressivity. The scoring system to express the ki67 proliferation index is based on the percentage of stained tumour cells by an antibody acting against the ki67 antigen. The commonly used antigen to stain formalin-fixed paraffin-embedded tissue samples is the ki67/MIB-1 (Mind Bomb Homolog 1) monoclonal antibody. The neoplasms are classified as low, intermediate and high in terms of their proliferative ability, corresponding to percentages of ≤15%, 16–30% and >30%, respectively [2]. To obtain better predictions of the disease outcome in thyroid carcinoma, many immunohistochemical markers have been studied to find suitable tools; ki67 is considered to be a promising tool for evaluating cell proliferation. It may have an important predictive role regarding the disease-free survival, disease-specific survival and prognosis of patients with DTC [14].

This narrative literature review aims to shed light on the role that ki67 may play in managing patients with DTC according to the latest evidence.

## 2. Materials and Methods

We performed a search in the PubMed and Goggle Scholar international databases using the following controlled keywords: “Ki 67”, “thyroid”, “carcinoma”, “differentiated”, “papillary”, “follicular” and “English publications”. The search was carried out using the controlled keywords included in three syntaxes: “ki 67 in differentiated thyroid carcinoma”, ki 67 in papillary thyroid carcinoma” and “ki 67 in follicular thyroid carcinoma” for a comprehensive search on the topic of ki67 and DTC association. The search process and the article selection/analysis were performed during January 2024 and April 2024 and targeted the articles from the earliest records to April 2024.

The inclusion criteria were articles pointing out the ki67 relationship with differentiated thyroid carcinoma, full-text articles on the topic of interest and English publications.

The exclusion criteria were articles not directly related to ki67 expression and to differentiated thyroid carcinoma and articles published before 2000.

When the potentially eligible papers were retrieved, the full-text articles were analysed for their eligibility. Thus, 68 items were identified. Another 5 items were identified by a hand search and were added to the records database. A total number of 73 records were considered for analysis. We proceeded to identify the duplicated articles, and the number of duplicates was none. We refined the selection, only considering eligible articles consisting of full-text original articles and literature reviews. Thus, 61 articles were considered eligible, and 32 articles were excluded because the content was not directly related to ki67 expression and/or to differentiated histological types of thyroid carcinoma, or they were published before 2000.

**Figure 1:**
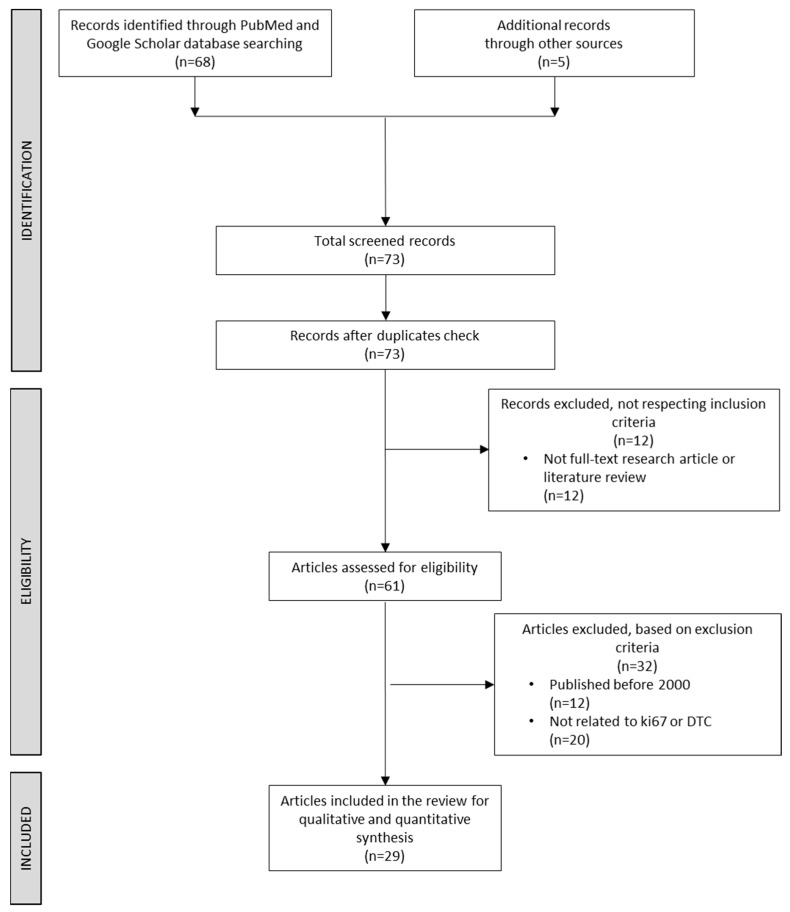
Selection criteria (PRISMA Flow Diagram).

After the selection criteria were respected and exclusion criteria were applied, a total number of 29 articles were included in this literature review, as presented in Figure 1.

## 3. Results and Discussion

Most of the studies included in the present review consist of retrospective studies (89.66%), and only a few case reports (6.9%) and literature reviews (3.45%) were selected as eligible. The studies evaluate the role and implications of ki67 solely or in comparison with other parameters as a diagnostic and/or prognostic tool regarding differentiated thyroid carcinoma. However, a proportion of 27.59% of the research papers included cases of anaplastic or medullary thyroid carcinoma even if the targeted cases set as inclusion criteria are represented by differentiated thyroid carcinoma. The results, conclusions and discussion related to these histological types are not presented or discussed in the present literature review.

The studies included in the present review are listed in Table 1.

Many statistical correlations and analyses have been conducted between ki67 and other genetic, molecular, histopathological and clinical features, with the purpose of improving the prognostic and predictive value of the entire panel of tools used by decision makers when the treatment strategies are elaborated on.

### 3.1. The ki67 Proliferation Index in Relation to Genetic and Molecular Features

BRAF (B-Raf proto-oncogene and serine/threonine kinase) mutations are some of the most frequent genetic alterations in well-differentiated thyroid carcinoma, as Harahap et al. stated in their article [15]. BRAF V600E represents the most frequent mutation of the BRAF proto-oncogene in its activation segment, targeting the V600 amino acid residue and resulting in a strong activation of BRAF kinase activity, the consequences being an increased proliferation rate and cellular growth [16]. In their study on 40 cases of recurrent well-differentiated thyroid carcinoma, Harahap et al. [15]. found that the BRAF V600E intensity was not correlated with the disease recurrence. The patients with strong BRAF V600E intensity had an average disease-free survival of 48 months (95% CI 40–56 months) compared to those with moderate BRAF V600E intensity. The latter had an average disease-free survival of 60 months (95% CI 35–63 months), and the statistical analysis showed no relationship between the BRAF V600E expression intensity and thyroid carcinoma recurrence (*p* = 0.661). The same study revealed a strong correlation between the ki67 expression (at least equal with 15 stained tumour cells) and the disease recurrence since the group of individuals with positive ki67 expression experienced an average disease-free survival of 40 months (95% CI 35–45 months) in comparison with the group of individuals with negative ki67 expression (less than 15 stained tumour cells), who experienced an average disease-free survival of 60 months (95% CI 53–67 months). This is based on the strongly correlated data between the ki67 expression and disease recurrence (*p* = 0.008) [15]. The authors concluded that the ki67 expression was correlated with the recurrence risk in well-differentiated thyroid carcinoma based on its main characteristics and was associated with cell proliferation and tumour growth. They also affirmed that, for a stronger predictive value of the disease-free survival, ki67 could be associated with other molecular markers like BRAF V600E [15].

Tang et al. published another interesting association in their research study performed on 42 cases of papillary thyroid carcinoma revealing the benefit of combining the loss of retinoid receptors evaluation with ki67 for the improved identification of aggressive papillary thyroid carcinomas. Retinoic acids are metabolites of vitamin A, which regulates the differentiation, proliferation and morphogenesis processes, and their effects are transduced by the retinoic acid nuclear receptors (RARα, RARβ, and RARγ) and retinoid X nuclear receptors (RXRα, RXRβ, and RXRγ) [17]. Tang et al. found that the loss of expression was met in 7.1% of the cases for RARα, 38.9% of the cases for RARβ, 18.2% of the cases for RXRα, 15.6% of the cases for RXRβ and in none for RARγ or RXRγ; a total number of 18 from 42 cases (42.9%) lost their expression of retinoid nuclear receptors. The ki67 proliferation index (the percentage of the stained tumour cells to the total counted tumour cells) evaluated in this study on all 42 cases had a mean value of 2.6 ± 1.6, ranging from 0.7 to 7.5%; the cases were divided into three groups by the level of ki67 proliferation index (0–2% ki67 index, 2–5% ki67 index and >5% ki67 index). The loss of expression for the retinoid receptors occurred more frequently in the cases of papillary thyroid carcinoma in the groups where the ki67 value was higher [17]. In the article published by Tang et al., the authors suggested that the information about the loss of expression for retinoid receptors might be used to identify the more aggressive cases of papillary thyroid carcinoma with high KI67 proliferation index values while losing their cellular differentiation [17].

The biological behaviour of papillary and follicular-derived carcinomas is often mild, but they may be partly differentiated, or may become anaplastic (dedifferentiated) when they grow fast and become more aggressive than the usual type [18]. Ito et al. studied the RCAS1 (receptor-binding cancer antigen expressed on SiSo cells) expression for normal thyroid cells, follicular thyroid adenoma and carcinoma, papillary thyroid carcinoma and undifferentiated thyroid carcinoma in 141 cases. RCAS1 is a membrane protein present in some carcinoma types. It plays a role in tumour immune evasion by counterattacking cytotoxic T lymphocytes and natural killer cells. This way, tumour cells are able to evade death, which would be induced by apoptosis [18]. The study revealed that the overexpression of RCAS1 was identified in 62.5% of the undifferentiated thyroid carcinomas, in 33.3% of the poorly differentiated thyroid carcinomas and 12.3% of the well-differentiated thyroid carcinoma cases. The authors found that the ki67 index (counted positive stained cells from at least 300 counted cells in three fields) increased with the grade of dedifferentiation, 45.5 ± 12.6 for undifferentiated cases in comparison with 2.3 ± 1.7 in well-differentiated cases. However, Ito et al. found no correlation between the ki67 index and RCAS1 overexpression [18].

In another study by Ito and his co-workers on 149 patients, the overexpression of PLK1 (polo-like kinase 1) in different types of thyroid carcinomas was evaluated. The polo-like kinase 1 is a protein playing a role in cell cycle regulation and, as Holtrich et al. and Yuan et al. demonstrated, it is a marker of cell proliferation [19]. Ito et al. found that the co-expression of PLK1 and the ki67 labelling index is rarely observed in papillary thyroid carcinoma, concluding that PLK1 does not play a role in cell proliferation and is not related to the tumour growth and does not contribute directly to the mitosis of papillary neoplastic cells [19].

The malignant cells are associated with a high metabolic rate, requiring a substantial amount of energy in comparison to normal tissues. In cancer cases, it has been observed that the overexpression of the GLUT1 cell membrane in particular, one member of the glucose transporters family (GLUT1-12), is responsible for the high glucose transport levels addressing the energetic needs of the neoplastic cell [20]. Grabellus et al. evaluated the correlation between the GLUT1 transporter overexpression and tumour proliferation in thyroid carcinomas, these immunohistochemical analyses also being correlated with the F-18-FDG PET/CT and I-124 PET/CT imaging studies in research on 181 subjects [20]. They found a clear correlation between the GLUT1 expression and ki67 index for all the types of thyroid carcinomas. The mean ki67 index values (expressed as the number of immunostained cells per high power field) were 10 ± 10 in the GLUT1 negative group, 46 ± 59 in the group with weak GLUT1 expression, 89 ± 87 in the group with intermediate GLUT1 expression and 122 ± 54 in the group with strong GLUT1 expression [20]. On the contrary, in a study by Schönberger et al., performed on 45 subjects, no significant relationship between the ki67 index and GLUT1 expression was found for differentiated tumours in contrast with anaplastic carcinoma cases according to the authors’ opinion. However, the data are not shown in the article, and the KI67 index was evaluated only for 22 of the patients [21].

Letsas et al. presented a research study on the correlations between proliferation and apoptosis in benign and malignant thyroid tissues. The study evaluates the immuno-expression of several experimental parameters like ProTα (Prothymosin α), E2F-1 transcription factor, p53 tumour suppression protein, BcL2 protein and Bax protein [22]. ProTα is an acidic nuclear protein that plays a vital role in the cell cycle regulation and cell proliferation in normal and malignant cells [22]. Its expression is upregulated mainly by the E2F-1 transcription factor and downregulated by the p53 tumour suppression protein [22]. ProTα may play the role of an anti-apoptotic factor [22]. The E2F-1 transcription factor may force the cells from the quiescent phase to pass into the S cellular phase and has been reported to be a neoplastic inductor for fibroblast cells, being found in elevated levels in breast, lung, gastrointestinal and thyroid carcinomas [22]. The p53 tumour suppressor protein is responsible for the cell arrest in the G1 phase when DNA (deoxyribonucleic acid) damage occurs, until DNA repair is realized or when the DNA damage is so harmful to the cell that p53 induces apoptosis mediated via BcL2/Bax proteins; Bcl2 promotes cell survival and Bax is responsible for cell death induction [22]. The study performed by Letsas et al. revealed that the ki67 proliferation index (expressed as a percentage of positive stained cells in the total number of counted cells) had mean values of 0.24 ± 0.11 in normal thyroid tissue, 0.74 ± 1.08 in thyroid nodular goitre, 0.96 ± 0.59 in follicular adenoma, 3.30 ± 2.00 in papillary thyroid carcinoma and 3.88 ± 2.77 in follicular thyroid carcinoma. The study showed a significant variation regarding the proliferation index/apoptotic index ratio (PI/AI) between several histological groups: 9.7 and 9.4 in follicular thyroid carcinoma and papillary thyroid carcinoma, respectively, 3.4 and 4.1 in follicular adenoma and nodular goitre, respectively, and 1.8 for normal thyroid tissue [22]. The authors concluded that the PI/AI index might be used as a diagnostic tool, especially in discriminating follicular neoplasia [22].

Hellgren et al. compared the ki67 proliferation index higher than 4% with the TERT (telomerase reverse transcriptase) promoter mutation status and with the presence of TERT gene expression, and they found a clear correlation between the ki67 proliferation index and TERT promoter mutation (*p* = 0.04), and also between the ki67 proliferation index and TERT gene expression (*p* < 0.001) [23].

The relationships between the ki67 proliferation index and the other genetic and molecular features identified in the reviewed articles are summarized in Table 2.

### 3.2. The ki67 Proliferation Index in Relation to Clinico-Pathological Features

Tan et al. performed a study on 39 cases of papillary thyroid carcinoma [17], follicular carcinoma [7] and follicular adenoma [15] to evaluate the differences or associations among several immunohistochemical markers used in differentiated follicular epithelial neoplasms. Their results were correlated with clinico-pathological risk factors. The ki67 proliferation index expressed as a proportion of nuclear-positive staining cells had no significant correlation with age, sex and tumour size for any of the histological types [24]. The results of the ki67 proliferation index they reported were statistically significant between papillary thyroid carcinoma and follicular carcinoma (*p* = 0.038) and between papillary thyroid carcinoma and follicular adenoma (*p* = 0.008), but no statistical significance was observed between follicular adenoma and follicular carcinoma (*p* = 0.739) [24].

A newly introduced histological subtype is differentiated high-grade thyroid carcinoma (DHGTC), a follicular-cell-derived lesion presenting well-differentiated areas with >5 mitosis per 2 mm^2^ and/or necrosis, most of them being papillary carcinomas with aggressivity signs [25]. Resta et al. published a research study on 32 patients with DHGTC and reported an average value of the ki67 proliferation index of 5.6%, with a median of 3.5% (range 1–20%) in 22 cases [25]. Another study from Thompson et al. reported ki67 proliferation index values calculated for DHGTC ranging from 2.3 to 19.6%, with a median of 8.3% and an average of 9.4% in their study performed on 41 subjects [26]. In their case study of DHGTC, Murata et al. reported a ki67 proliferation index value of 10%, higher than the aforementioned value [27].

Tang et al. obtained good correlations between the ki67 proliferation index and some histopathological features of the 42 cases of PTC included in their study. They analysed the histopathological characteristics of the subjects included and searched for correlations with the ki67 proliferation index. Characteristics like trabecular, solid or scirrhous growth pattern, cellular polarity and loss of cohesiveness were correlated with a high ki67 index value (2–5% ki67 and >5%, respectively). The capsulated tumours were met in the low ki67 group (<2%), and the infiltrative growth pattern was met in the highest ki67 group (>5%). They found no statistically significant difference regarding ki67 correlated with age, sex, stage and nodal metastatic involvement [17]. Converging to these results, in one study regarding the overexpression of RCAS1 in DTC, Ito et al. also concluded that the ki67 proliferation index significantly increases with dedifferentiation [18].

In their study on a columnar subtype of papillary thyroid carcinoma, despite the recognized fact that the presence of columnar cells represents a sign of tumour aggressiveness [11], Sujoy et al. concluded in their analysis of 10 patients that there was no relationship between the ki67 proliferation index and the biological behaviour of the tumour [28].

In their already mentioned study, Letsas et al. also found that there was a significant difference between the papillary and follicular carcinomas in comparison with normal thyroid tissue, nodular goitre or follicular adenoma. They found significantly higher ki67 proliferation index values for papillary thyroid carcinoma and follicular thyroid carcinoma (mean 3.3% ± 2, range 1–7; mean 3.88 ± 2.77, range 1–8, respectively) [22]. Another interesting result was published by Poloz et al., who, in their research on 266 patients concerning the differential diagnosis of follicular adenoma, follicular thyroid carcinoma and papillary thyroid carcinoma, found that the ki67 proliferation index is significantly higher in papillary thyroid carcinoma (34.1 ± 1.3) but without significant differences in follicular thyroid carcinoma compared with follicular adenoma and atypical follicular adenoma (6.6 ± 1.19, 8.2 ± 0.2 and 7.4 ± 0.1, respectively) [29]. Another study on the Chinese population published by Song et al. affirmed that there is no statistical significance regarding ki67 in papillary thyroid carcinoma compared with benign thyroid lesions [30].

In a study by Nasr et al., the results revealed good cytoplasmic ki67 immunostaining for papillary thyroid carcinoma cells in comparison with non-neoplastic thyroid lesions [31], but these results were obtained only over a small percentage of the subjects, and the ki67 index was not included in the entirety of the research [31].

Ozolins et al. concluded in their study that the ki67 index was generally low <5% but higher in PTC than in the surrounding tissue (2.36 ± 0.85) and higher in FTC than in the colloid goitre and follicular adenoma (3.62 ± 1.1) [32].

The study of Saini et al. did not find ki67 expression on benign tissues, low staining rates for benign lesions and an increased proportion of staining regarding PTC cases, the mean values for metastatic PTC being significantly different from other histologic categories (*p* < 0.001) [33].

Grabellus et al. affirmed in their study that the ki67 index increased with the decreasing differentiation of the respective tumour type, the mean values of the ki67 proliferation index expressed as the number of positive nuclei per high-power field being 6 ± 7 for thyroid adenoma, 16 ± 19 for follicular thyroid carcinoma, 12 ± 10 for papillary thyroid carcinoma, 71 ± 68 for poorly differentiated thyroid carcinoma and 144 ± 60 for anaplastic thyroid carcinoma [20].

Dwidedi et al. published results showing that the differences in the ki67 index mean were statistically significant between papillary thyroid carcinoma (3.45 ± 2.40) and follicular adenoma (*p* < 0.05), follicular adenoma (2.23 ± 1.05) versus follicular carcinoma (*p* < 0.05), follicular variant of papillary carcinoma (3.34 ± 2.04) versus follicular adenoma (*p* < 0.05) and malignant tumours (3.47 ± 2.33) and non-malignant lesions/tumours (1.37 ± 0.94) (*p* < 0.001) [34].

Pujani et al. revealed the results of a study on 100 subjects showing that the ki67 proliferation index mean was the highest for undifferentiated carcinoma (9.0), followed by medullary carcinoma (7.0), follicular carcinoma (6.0) and papillary carcinoma (3.66) and the non-neoplastic disease (0.21) [35].

Strong ki67 staining showing a high proliferative rate was revealed by Maia et al. in their study, where the highest proliferative rate was obtained in papillary thyroid carcinoma cases (34%), followed by follicular thyroid adenoma (19%), Hashimoto thyroiditis (18.8%), adenomatoid hyperplasia (7.1%) and normal thyroid tissues (*p* = 0.031) [36].

Müssig et al. found that the ki67 proliferation index was significantly correlated with the tumour staging (stage I 5% ± 3; stage II 6% ± 5; stage III 7% ± 5; stage IV A 7% ± 4 and stage IV C 9% ± 5; *p* = 0.0459). Their analysis also revealed that the tumour had an aggressive nature when the ki67 index was greater than 15%, whereas the disease showed a benign course when the ki67 index was under 5% [37].

Gupta et al. exploited a different perspective. They conducted research trying to evaluate the impact of the lymphocytic infiltration of DTC by proliferating lymphocytes on the patient outcome. For this, they examined and immunostained the adjacent sections in 63 cases, identifying the presence of tumour-associated lymphocytes (expression of leucocyte common antigen LCA) and their proliferation status (expression of ki67). They reported the number of LCA-positive lymphocytes and ki67-positive lymphocytes over four categories of cases (papillary thyroid carcinoma, follicular thyroid carcinoma, autoimmune thyroid disease and nonimmune thyroid disease). They found that the LCA expression had the greatest values in the autoimmune thyroid disease (50.5 ± 20.6 cells) and was significantly higher in PTC (32.2 ± 7.5) compared with FTC (5.6 ± 2.5) or nonimmune disease (1.4 ± 1.2). The greatest ki67 expression was found in PTC (13.9 ± 4.8) compared with FTC (4.7 ± 2.3) or nonimmune thyroid disease (0.39 ± 0.28) [38].

Hellgren et al. found in their study on 818 subjects that the ki67 proliferation index had an average value of 5.8% (1–32) in follicular thyroid carcinoma cases, 5.1% (1–14) in follicular tumours of uncertain malignant potential and 2.6% (0.5–17) in follicular adenomas, significantly higher in follicular thyroid carcinoma cases in comparison with follicular adenoma cases (*p* < 0.001) but not higher than in cases of follicular tumours of uncertain malignant potential (*p* = 0.3) [23].

The histological types of DTC assessed by ki67 and mentioned in the reviewed articles are listed in Table 3.

### 3.3. The ki67 Proliferation Index as a Prognostic Tool

Harahap et al. found that the ki67 expression and recurrence of thyroid cancer can be associated based on disease-free survival, which has a hazard ratio of 1.34 (1.13–1.92), and concluded that the ki67 proliferation index can be a predictor of thyroid cancer recurrence [15].

Thompson et al. mentioned in their study that there was a trend to higher ki67 proliferation indices, suggesting metastatic disease development [26].

Kirdak et al. concluded in their study on three cases of thyroid carcinoma with insular components that the ki67 proliferation index may be useful in predicting morbidity and mortality and may also be useful in categorising cases with insular components in subgroups for better risk stratification and prognosis [39].

Sujoy et al. found no correlation between the ki67 index and the biological behaviour of the tumours; their study was aimed at a columnar variant of PTC and performed on ten patients [28].

An interesting finding was presented by Gupta et al. in their study related to tumour-associated lymphocytes in DTC occurring in children and young adults. They mentioned that the ki67 proliferating index for tumour-associated lymphocytes in DTC is important in predicting recurrence for young patients since the tumours expressing proliferating associated lymphocytes have a significantly reduced risk of recurrence [38].

Müssig et al. tried to evaluate the association of ki67 with the tumour staging and clinical outcome of DTC and concluded in their research that the ki67 proliferation index represents a predictor for the clinical outcome at five years after definitive treatment in DTC (*p* < 0.0001). Since there is a difference between the clinical outcomes of papillary and follicular thyroid carcinomas, they evaluated both entities separately and found that the ki67 index can be a predictor of the clinical outcome five years after the definitive treatment for the papillary type (*p* < 0.0001), in contrast with the follicular type [37].

In their study on papillary thyroid carcinoma, Radu et al. associated the low expression of ki67 with the favourable evolution of the disease after treatment [40].

Hellgren et al. presented in their study on 818 subjects a survival analysis revealing that the optimal cutoff for the ki67 proliferation index for follicular thyroid carcinoma is 4%, a cutoff value that would split the subjects into two risk categories in terms of metastatic development/recurrence and also in terms of death from disease, with a sensitivity of 80% and a specificity of 48%. The difference between the group with ki67 > 4% and the group with ki67 ≤ 4% was statistically significant, with *p* < 0.001 for metastatic development/recurrence and *p* = 0.005 for death caused by the disease. This provides ki67 a predictive value for metastatic events/recurrence and death caused by the disease, the values > 4% being significantly associated with poorer prognostic and metastatic events or recurrence. Moreover, ki67 has a prognostic value that is independent of the tumour size since, for each pT1, pT2 or pT3 category, regarding the comparison between the ki67 ≤ 4% and ki67 > 4% groups, the authors found significant differences in the disease-free-survival interval (*p* = 0.011, *p* = 0.046 and *p* = 0.002, respectively). Since for pT4 all the lesions had ki67 > 4%, no comparison was performed [23].

Mu et al. found that the ki67 proliferation index determined on specimens of fine-needle aspiration biopsy may be a predictive factor for follicular thyroid carcinoma since the preoperative distinction between the two entities—follicular thyroid carcinoma and follicular thyroid adenoma—can be a real challenge and ki67 proliferation may add diagnostic value [41].

Lindfors et al. evaluated the predictive value of ki67 determined for the primary tumour and for the metastatic lymph nodes in a study on 327 cases related to the recurrence risk in relation to cytoplasmatic Tiroglobulin (Tg) expression for papillary thyroid carcinoma. The value of ki67 ≥ 2.45% in the primary tumour has a predictive value for tumour recurrence with a sensitivity of 68% and specificity of 56%. The value of ki67 ≥ 2.85% in the lymph nodes is a predictor of tumour recurrence with a sensitivity of 76% and a specificity of 52%. The authors also evaluated the correlation of ki67 with Tg expression and found that Tg was inversely correlated with ki67 and related to the recurrence. The patients with lymph node metastases with ki67 < 2.85% and Tg expression > 75% had longer recurrence-free survival (153 months) in comparison with the patients with Tg: 0–75% (130 months), the difference being statistically significant (*p* + 0.019) [42].

The prognostic value of ki67 and the prognostic indicators are pointed out in Table 4.

## 4. Conclusions

The studies included in the present review presented converging data regarding most of the aspects concerning the ki67 proliferation index. Thus, several results showed slight differences.

The ki67 proliferation index in differentiated thyroid carcinoma is a diagnostic/prognostic tool of interest, which should be exploited to its maximum extent.

It is clear that large cohorts of subjects and prospective studies may produce more consistent and accurate data, resulting in superior value for ki67 as a specific tool in the management strategy of differentiated thyroid carcinoma, with clear indications and pertinent cutoff values, since, at present, these aspects are peculiar and not fully standardized.

The ki67 proliferation index is a good predictor of disease-free survival, disease recurrence and metastatic development, being strongly correlated with the mortality and morbidity, tumour dedifferentiation and tumour aggressiveness in differentiated thyroid carcinoma.

In light of the reviewed literature, we affirm that the ki67 proliferation index may contribute to valuable outcomes if introduced in the histopathological reports of differentiated thyroid carcinoma since it is not presently a routine practice.

## Figures and Tables

**Table 1:** Studies included in the review.

NrCrt	First Author’s Name	Year of Publication	Study Type	No. of Subjects
1	Gupta S. **, ^c^	2001	retrospective	63
2	Schönberger J. *, ^p^	2002	retrospective	45
3	Tang W. ^p^	2003	retrospective	42
4	Ito Y. * ^p^	2003	retrospective	141
5	Ito Y. * ^p^	2004	retrospective	149
6	Letsas K. P. ^p^	2005	retrospective	40
7	Nasr M. R. ^p^	2006	retrospective	108
8	Kirdak T. ^c^	2006	case report	3
9	Poloz T. L. ^p^	2008	retrospective	266
10	Ozolins A. ^p^	2010	retrospective	148
11	Pujani M. *,**, ^p^	2010	retrospective	100
12	Song Q. ^p^	2011	retrospective	592
13	Tan A. ^p^	2011	retrospective	39
14	Müssig K. ^p^	2012	retrospective	93
15	Grabellus F. *, ^c^	2012	retrospective	181
16	Sujoy V. ^p^	2013	retrospective	10
17	Hayam A. ^p^	2013	retrospective	80
18	Saini L. M. ^p^	2015	retrospective	26
19	Maia F. F. R. ^p^	2015	retrospective	282
20	Radu T. G. ^p^	2015	retrospective	27
21	Kakudo K. *, ^c^	2015	review	NAP
22	Dwidedi S. S. ^p^	2016	retrospective	160
23	Mu N. ^c^	2018	retrospective	61
24	Harahap W. A. ^c^	2022	retrospective	40
25	Hellgren S. L. ^c^	2022	retrospective	818
26	Thompson L. D. R. ^c^	2023	retrospective	41
27	Murata S. ^p^	2023	case report	1
28	Lindfors H. ^c^	2023	retrospective	327
29	RestaT. I. ^c^	2024	retrospective	32

* the research included anaplastic thyroid carcinoma cases; ** the research included medullary thyroid carcinoma cases; ^p^ the research aims for preclinical data; ^c^ the research aims for clinical data.

**Table 2:** The relationship between the ki67 proliferation index and other genetic and molecular features [15,17,18,19,20,21,22,23].

NrCrt	First Author’s Name	Year of Publication	Related Genetic or Molecular Feature	Correlation with ki67 Proliferation Index
1	Schönberger J. [21]	2002	GLUT1	No correlation
2	Tang W. [17]	2003	Retinoid receptor status	Loss of expression to identify the more aggressive cases of PTC with high ki67 proliferation index
3	Ito Y. [18]	2003	RCAS1	Uncorrelated with ki67 proliferation index
4	Ito Y. [19]	2004	PLK1	Co-expression rarely seen
5	Letsas K. P. [22]	2005	Apoptotic index	PI/AI may be used as a diagnostic tool
6	Grabellus F. [20]	2012	GLUT1	Clear correlation with ki67 proliferation index
7	Harahap W. A. [15]	2022	BRAF V600E	Impoves the prediction outome of ki67 proliferation index, especially in discriminated thyroid carcinoma
8.	Hellgren L. S. [23]	2022	TERT promoter mutation and TERT gene expression	Clear correlation with ki67 proliferation index

**Table 3:** Histological types [17,20,22,23,24,25,28,29,30,31,32,33,34,35,36,37,38].

NrCrt	First Author’s Name	Year of Publication	Histological Type
1	Gupta S. [38] **,***	2001	Papillary thyroid carcinoma, Follicular thyroid carcinoma *
2	Tang W. [17]	2003	Papillary thyroid carcinoma
3	Letsas K. P. [22]	2005	Papillary thyroid carcinoma, Follicular thyroid carcinoma
4	Nasr M. R. [31]	2006	Papillary thyroid carcinoma
5	Poloz T. L. [29]	2008	Follicular thyroid carcinoma, Papillary thyroid carcinoma
6	Ozolins A. [32]	2010	Papillary thyroid carcinoma, Follicular thyroid carcinoma
7	Pujani M. [35] *,**	2010	Papillary thyroid carcinoma, Follicular thyroid carcinoma
8	Song Q. [30]	2011	Papillary thyroid carcinoma
9	Tan A. [24]	2011	Papillary thyroid carcinoma, Follicular thyroid carcinoma
10	Müssig K. [37]	2012	Papillary thyroid carcinoma, Follicular thyroid carcinoma
11	Grabellus F. [20] *	2012	Papillary thyroid carcinoma, Follicular thyroid carcinoma, Poorly differentiated thyroid carcinoma
12	Sujoy V. [28]	2013	Papillary thyroid carcinoma columnar subtype
13	Saini L.M. [33]	2015	Papillary thyroid carcinoma
14	Maia F. F. R. [36]	2015	Papillary thyroid carcinoma
15	Dwidedi S. S. [34]	2016	Papillary thyroid carcinoma, Follicular thyroid carcinoma, Follicular variant of papillary thyroid carcinoma
16	Hellgren L. S. [23]	2022	Follicular thyroid carcinoma, Follicular tumour of uncertain malignant potential
17	Resta T.I. [25]	2024	Differentiated high-grade thyroid carcinoma

* the research included anaplastic thyroid carcinoma cases; ** the research included medullary thyroid carcinoma cases; *** presence of LCA.

**Table 4:** The prognostic indicators and the correlation with ki67 [15,23,26,28,37,38,39,40,41,42].

NrCrt	First Author’s Name	Year of Publication	Prognostic Indicator	Correlation with ki67
1	Gupta S. [38]	2001	Recurrence	Yes
2	Kirdak T. [39]	2006	Morbidity, Mortality	Yes
3	Müssig K. [37]	2012	Clinical outcome at 5 years after definitive treatment	Yes
4	Sujoy V. [28]	2013	Tumour biological behaviour	Yes
5	Radu T. G. [40]	2015	Favourable evolution	Yes *
6	Mu N. [41]	2018	Follicular thyroid carcinoma	Yes **
7	Harahap W. A. [15]	2022	Recurrence	Yes
8	Hellgren L. S. [23]	2022	Metastatic development, Recurrence, Death of disease	Yes
9	Thompson L. D. R. [26]	2023	Metastatic disease development	Yes
10	Lindfors H. [42]	2023	Recurrence	Yes

* inverse relation; ** ki67 is determined in preoperative fine-needle aspiration biopsy.

## Data Availability

The authors confirm that the data supporting this study’s findings are available within the article.

## References

[1] Norton L. (2014). Cancer Log-Kill Revisited. Am. Soc. Clin. Oncol. Educ. Book.

[2] Li L.T., Jiang G., Chen Q., Zheng J.N. (2015). Ki67 is a promising molecular target in the diagnosis of cancer (Review). Mol. Med. Rep..

[3] Lee T.-K., Myers R.T., Marshall R.B., Bond M.G., Kardon B. (1985). The significance of mitotic rate: A retrospective study of 127 thyroid carcinomas. Hum. Pathol..

[4] Menon S.S., Guruvayoorappan C., Sakthivel K.M., Rasmi R.R. (2019). Ki-67 protein as a tumour proliferation marker. Clin. Chim. Acta.

[5] Viana A.d.O.R., Filho J.G., Francisco A.L.N., Pinto C.A.L., Kowalski L.P. (2020). ki67 and ck-19 are predictors of locoregional recurrence in papillary thyroid carcinoma. Acta Otorhinolaryngol. Ital..

[6] Dencic T.I., Cvejic D., Paunovic I., Tatic S., Havelka M., Savin S. (2012). Cytokeratin19 expression discriminates papillary thyroid carcinoma from other thyroid lesions and predicts its aggressive behavior. Med. Oncol..

[7] Kitahara C.M., Sosa J.A. (2016). The changing incidence of thyroid cancer. Nat. Rev. Endocrinol..

[8] O’Grady T.J., Gates M.A., Boscoe F.P. (2015). Thyroid cancer incidence attributable to overdiagnosis in the United States 1981–2011. Int. J. Cancer.

[9] Piciu D., Irimie A. (2007). Diagnosis and Treatment Guidlines in Thyroid Carcinoma. American and European Consensus, adapted to Romania. Acta Endocrinol..

[10] Gaengler S., Andrianou X., Piciu A., Charisiadis P., Zira C., Aristidou K., Piciu D., Makris K. (2017). Iodine status and thyroid nodules in females: A comparison of Cyprus and Romania. Public Health.

[11] Jung C.K., Bychkov A., Kakudo K. (2022). Update from the 2022 World Health Organization Classification of Thyroid Tumors: A Standardized Diagnostic Approach. Endocrinol. Metab..

[12] Sun X., Kaufman P.D. (2018). Ki-67: More than a proliferation marker. Chromosoma.

[13] Urruticoechea A., Smith I.E., Dowsett M. (2005). Proliferation marker ki67 in early breast cancer. J. Clin. Oncol..

[14] Miyauchi A., Kudo T., Hirokawa M., Ito Y., Kihara M., Higashiyama T., Yabuta T., Masuoka H., Shindo H., Kobayashi K. (2013). Ki-67 Labeling Index Is a Predictor of Postoperative Persistent Disease and Cancer Growth and a Prognostic Indicator in Papillary Thyroid Carcinoma. Eur. Thyroid. J..

[15] Harahap W.A., Tofrizal T., Oktahermoniza O. (2022). Relationship between the Expression of BRAF V600E and Ki-67 with the Recurrence of Well-Differentiated Thyroid Cancer. Asian Pac. J. Cancer Prev..

[16] Consoli F., Barbieri G., Picciolini M., Medicina D., Bugatti M., Tovazzi V., Liserre B., Zambelli C., Zorzi F., Berruti A. (2020). A Rare Complex BRAF Mutation Involving Codon V600 and K601 in Primary Cutaneous Melanoma: Case Report. Front. Oncol..

[17] Tang W., Nakamura Y., Zuo H., Yasuoka H., Yang Q., Wang X., Nakamura M., Mori I., Miyauchi A., Kakudo K. (2003). Differentiation, proliferation and retinoid receptor status of papillary carcinoma of the thyroid. Pathol. Int..

[18] Ito Y., Yoshida H., Nakano K., Kobayashi K., Yokozawa T., Hirai K., Matsuzuka F., Matsuura N., Kuma K., Miyauchi A. (2002). Overexpression of human tumor-associated antigen, RCAS1, is significantly linked to dedifferentiation of thyroid carcinoma. Oncology.

[19] Ito Y., Miyoshi E., Sasaki N., Kakudo K., Yoshida H., Tomoda C., Uruno T., Takamura Y., Miya A., Kobayashi K. (2004). Polo-like kinase 1 overexpression is an early event in the progression of papillary carcinoma. Br. J. Cancer.

[20] Grabellus F., Nagarajah J., Bockisch A., Schmid K.W., Sheu S.-Y. (2012). Glucose transporter 1 expression, tumor proliferation, and iodine/glucose uptake in thyroid cancer with emphasis on poorly differentiated thyroid carcinoma. Clin. Nucl. Med..

[21] Schönberger J., Rüschoff J., Grimm D., Marienhagen J., Rümmele P., Meyringer R., Kossmehl P., Hofstaedter F., Eilles C. (2002). Glucose transporter 1 gene expression is related to thyroid neoplasms with an unfavorable prognosis: An immunohistochemical study. Thyroid^®^.

[22] Letsas K.P., Frangou-Lazaridis M., Skyrlas A., Tsatsoulis A., Malamou-Mitsi V. (2005). Transcription factor-mediated proliferation and apoptosis in benign and malignant thyroid lesions. Pathol. Int..

[23] Hellgren L.S., Stenman A., Paulsson J.O., Höög A., Larsson C., Zedenius J., Juhlin C.C. (2022). Prognostic Utility of the Ki-67 Labeling Index in Follicular Thyroid Tumors: A 20-Year Experience from a Tertiary Thyroid Center. Endocr. Pathol..

[24] Tan A., Etit D., Bayol U., Altinel D., Tan S. (2011). Comparison of proliferating cell nuclear antigen, thyroid transcription factor-1, Ki-67, p63, p53 and high–molecular weight cytokeratin expressions in papillary thyroid carcinoma, follicular carcinoma, and follicular adenoma. Ann. Diagn. Pathol..

[25] Resta I.T., Gubbiotti M., Montone K., Livolsi V., Baloch Z. (2024). Differentiated high grade thyroid carcinomas: Diagnostic consideration and clinical features. Hum. Pathol..

[26] Thompson L.D.R. (2023). High Grade Differentiated Follicular Cell-Derived Thyroid Carcinoma Versus Poorly Differentiated Thyroid Carcinoma: A Clinicopathologic Analysis of 41 Cases. Endocr. Pathol..

[27] Murata S.I., Matsuzaki I., Kishimoto M., Katsuki N., Onishi T., Hirokawa M., Kojima F. (2023). Papillary thyroid carcinoma with aggressive fused follicular and solid growth pattern: A unique histological subtype with high-grade malignancy?. Pathol. Int..

[28] Sujoy V., Pinto A., Nosé V. (2013). Columnar cell variant of papillary thyroid carcinoma: A study of 10 cases with emphasis on CDX2 expression. Thyroid^®^.

[29] Poloz T.L., Shkurupiy V.A. (2008). Potentialities of differential immunohistochemical diagnosis of some follicular tumors of the thyroid gland. Bull. Exp. Biol. Med..

[30] Song Q., Wang D., Lou Y., Li C., Fang C., He X., Li J. (2011). Diagnostic significance of CK19, TG, Ki67 and galectin-3 expression for papillary thyroid carcinoma in the northeastern region of China. Diagn. Pathol..

[31] Nasr M.R., Mukhopadhyay S., Zhang S., Katzenstein A.L.A. (2006). Immunohistochemical markers in diagnosis of papillary thyroid carcinoma: Utility of HBME1 combined with CK19 immunostaining. Mod. Pathol..

[32] Ozolins A., Narbuts Z., Strumfa I., Volanska G., Gardovskis J. (2010). Diagnostic utility of immunohistochemical panel in various thyroid pathologies. Langenbeck’s Arch. Surg..

[33] Saini M.L., Weynand B., Rahier J., Mourad M., Hamoir M., Marbaix E. (2015). Cyclin D1 in well differentiated thyroid tumour of uncertain malignant potential. Diagn. Pathol..

[34] Dwivedi S.S., Khandeparkar S.G.S., Joshi A.R., Kulkarni M.M., Bhayekar P., Jadhav A., Nayar M., Kambale N.S. (2016). Study of immunohistochemical markers (CK-19, CD-56, KI67, p53) in differentiating benign and malignant solitary thyroid nodules with special reference to papillary thyroid carcinomas. J. Clin. Diagn. Res..

[35] Pujani M., Arora B., Singh S., Tejwani N. (2010). Role of Ki-67 as a proliferative marker in lesions of thyroid. Indian J. Cancer.

[36] Maia F.F.R., Vassallo J., Pinto G.A., Pavin E.J., Matos P.S., Zantut-Wittmann D.E. (2016). Expression of Mcl-1 and Ki-67 in Papillary Thyroid Carcinomas. Exp. Clin. Endocrinol. Diabetes.

[37] Müssig K., Wehrmann T., Dittmann H., Wehrmann M., Ueberberg B., Schulz S., Bares R., Petersenn S. (2012). Expression of the proliferation marker Ki-67 associates with tumour staging and clinical outcome in differentiated thyroid carcinomas. Clin. Endocrinol..

[38] Gupta S., Patel A., Folstad A., Fenton C., Dinauer C.A., Tuttle R.M., Conran R., Francis G.L. (2001). Infiltration of differentiated thyroid carcinoma by proliferating lymphocytes is associated with improved disease-free survival for children and young adults. J. Clin. Endocrinol. Metab..

[39] Kirdak T., Saraydaroglu O., Duran C., Yerci O., Korun N. (2006). Thyroid carcinoma with insular component: Report of three cases with different clinical pictures. Tumori J..

[40] Radu T.G., Mogoantă L., Busuioc C.J., Stănescu C., Grosu F. (2015). Histological and immunohistochemical aspects of papillary thyroid cancer. Rom. J. Morphol. Embryol..

[41] Mu N., Juhlin C.C., Tani E., Sofiadis A., Reihnér E., Zedenius J., Larsson C., Nilsson I.-L. (2018). High Ki-67 index in fine needle aspiration cytology of follicular thyroid tumors is associated with increased risk of carcinoma. Endocrine.

[42] Lindfors H., Karlsen M., Karlton E., Zedenius J., Larsson C., Lundgren C.I., Juhlin C.C., Shabo I. (2023). Thyroglobulin expression, Ki-67 index, and lymph node ratio in the prognostic assessment of papillary thyroid cancer. Sci. Rep..

